# Effects of heavy metals in acute ischemic stroke patients: A cross-sectional study

**DOI:** 10.1097/MD.0000000000028973

**Published:** 2022-03-04

**Authors:** Cheng-Chang Yen, Hsin-Hung Chen, Yi-Ting Hsu, Ching-Jiunn Tseng, Ching-Huang Lin

**Affiliations:** a Section of Neurology, Kaohsiung Veterans General Hospital, No. 386, Ta-Chung 1st Rd., Kaohsiung, Taiwan; b Department of Electrical Engineering, Southern Taiwan University of Science and Technology, No. 1, Nan-Tai Street, Yongkang Dist., Tainan, Taiwan; c Department of Medical Education and Research, Kaohsiung Veterans General Hospital, No. 386, Ta-Chung 1st Rd., Kaohsiung, Taiwan; d Department of Medical Research, China Medical University Hospital, China Medical University, No. 2, Yude Rd., Taichung, Taiwan; e Department of Biological Sciences, National Sun Yat-Sen University, 70 Lienhai Rd., Kaohsiung, Taiwan; f Department of Physical Therapy, Shu-Zen Junior College of Medicine and Management, No. 452, Huanqiu Rd. Luzhu Dist., Kaohsiung, Taiwan.

**Keywords:** environmental exposure, habits, health risks, heavy metals, stroke

## Abstract

Cerebrovascular disease is the second commonest cause of mortality globally and among the commonest causes of disability. However, research executed to probe the heavy metal exposure–stroke incidence relationship is scarce. Accordingly, we executed our study to probe the relationship of heavy metal concentrations (ie, concentrations of lead [Pb], mercury [Hg], cadmium [Cd], and arsenic) in the serum and urine of acute ischemic stroke (AIS) patients with several patient variables.

For enrollment, we chose patients who had a first AIS within 7 days after the onset of a stroke. Thus, 33 newly diagnosed patients with AIS were recruited. We determined the aforementioned metals’ concentrations by executing inductively coupled plasma mass spectrometry. We also gauged the association between such metal concentrations and patient variables by employing Spearman correlation coefficient. To examine the differences in metal concentrations between the different variables, we implemented an independent Mann–Whitney *U* test.

In our cohort analysis, we noted serum Pb and Cd concentrations to be positively correlated with serum creatinine and hemoglobin. Serum and urine Cd concentrations had a negative correlation with impaired HbA1c in AIS patients. Urine Hg had a positive correlation with C-reactive protein in the participants. Participants who smoked or consumed alcohol had significantly higher Pb and Cd levels in serum than did those who neither smoked nor drank. Patients with AIS who smoked or consumed alcohol had high levels of serum Pb and serum Cd than did those who did not. Patients with AIS who consumed alcohol had significantly higher Pb and Hg urine concentrations than did those who did not.

Our study indicated that serum Cd and Pb elevation increased the AIS risk in southern Taiwan patients.

## 1 Introduction

Statistics reveal that within the United States, stroke results in the death of over 140,000 persons yearly; this consequently renders it the third commonest cause of death in this nation. An estimated 6.6 and 2.5 million American and Chinese individuals, respectively, have a stroke every year.^[1,2]^ Worldwide, ≥5 million persons every year die of stroke; in addition, millions of people have long-term disabilities after a stroke.^[3]^ In Taiwan, cerebrovascular disease is the fourth commonest cause of mortality.^[4]^ Stroke is the most prevalent cerebrovascular event, with the commonest stroke event being ischemic stroke, occurring in approximately 85% of cases; additionally, hemorrhagic stroke, including cerebral and subarachnoid types, accounts for 15% of cases.^[1]^ Regarding acute ischemic stroke (AIS), examples of relevant risk factors are, but not limited to, male sex, age, hypertension, body mass index (BMI), diabetes, smoking status, hyperlipidemia, and alcohol consumption,^[5,6]^ which can be categorized as nonmodifiable (age, sex, and genetics) and modifiable (hypertension, diabetes, hyperlipidemia, poor diet, and smoking status). Clinical strategies aimed at managing modifiable risk factors can reduce the risk of AIS.

With recent industrialization and pollution of the environment, environmental toxins and heavy metals have been contributing to various conditions (eg, stroke).^[7,8]^ As demonstrated in the literature, exposure to arsenic (As), lead (Pb), cadmium (Cd), and mercury (Hg) – all of which constitute examples of heavy metal pollutants – is a major stroke risk factor.^[9,10]^ Associations of such pollutants with AIS risk factors remain an active research area. Exposure to immense As levels can engender peripheral artery disease development,^[11,12]^ such as black foot disease, which is a serious type of peripheral artery disease endemic to Taiwan.^[13]^ Exposure to Cd and Pb could heighten the stroke risk through the effects of smoking, increased oxidative stress, endothelial function alteration, the promotion of inflammation, the downregulation of nitric oxide generation, and a heightened risk of peripheral arterial disease.^[14]^ Hg, a trace metal, could be toxic to humans after exposure, which mainly occurs through the consumption of fish; such exposure has a significant association with stroke risk.^[6,15]^ Moreover, an association has been made between heavy metals and cigarette product and smoke toxicity. Cigarette smoke contains immense levels of Cd, As, and lead.^[16]^ In addition, smoking, including tobacco and electronic cigarettes, is linked to heighten sudden death risk.^[17]^ Smoking, alcohol consumption (excessive alcohol intake: >60 g per day), diabetes, dyslipidemia, and carotid artery disease as well as other heart diseases are modifiable stroke risk factors.^[18,19]^ Among the modifiable risk indicators, hypertension has the greatest impact on stroke risk.^[20]^ In fact, heavy metal pollution in the environment and lifestyle changes have increased the risk of AIS. However, evidence from longitudinal studies on modifiable AIS risk indicators and the effects of heavy metal pollution, particularly regarding the effects of combined environmental heavy metal pollution and lifestyle changes, is limited. Accordingly, the present study purpose was to use survey sampling to examine the impact of heavy metal (ie, Hg, As, Pb, and Cd) elevation on patients with AIS in southern Taiwan.

## 2 Methods

### 2.1 Study design and participants

Participants for our executed study comprised patients who received a first diagnosis of AIS occurring within a period of 3 to 7 days after a stroke and were enrolled in Kaohsiung Veterans General Hospital neurology department between August 2011 and March 2013. We adhered to all relevant international and US regulations, particularly the Declaration of Helsinki, for studies involving humans. Kaohsiung Veterans General Hospital Institutional Review Board approved this study (VGHKS 11-CT5-04). Participants provided informed consent at recruitment, and a questionnaire was administered at follow-up. If required, we gave participants assistance with questionnaire completion.

Patients were enrolled in our study only if they fulfill all of the following criteria: admission for first-event ischemic stroke; computed tomography or magnetic resonance imaging showing an acute ischemic lesion consistent with clinical manifestations verified by a neurologist; absence of any clinically relevant arrhythmia on admission, including atrial fibrillation; absence of any major concurrent diseases, including uremia, malignancies, and chronic obstructive pulmonary disease; and absence of fever, alternations in consciousness, or any relevant hemodynamic compromise on admission. Patients were excluded who were pregnant, were lactating, had intraparenchymal hemorrhage verified on brain images, had edetate calcium disodium (EDTA) sensitivity, had a stroke associated with a cardiac embolism or tumor, needed urgent surgical intervention, were in a critical condition in the ICU, were taking part in a different interventional study, or had given birth within 30 days prior to study execution.

### 2.2 Study procedures

Before concentrations of heavy metals were determined, all patients underwent neuroimaging, underwent complete blood count measurement, electrocardiogram examination, blood glucose measurement, and lipid and electrolyte profiling. To determine the presence of AIS, neuroimaging was applied through magnetic resonance imaging and computed tomography scans. A questionnaire was answered by each participant; questions included those on stroke risk factors, baseline characteristics, area of residence, and dietary habits.

### 2.3 Measurements of heavy metal concentrations

At baseline (2011–2013), following Lin et al,^[6]^ before infusing the chelating agent (EDTA), we detect the serum concentrations of As, Hg, Pb, and Cd. Twenty-four hours after the infusion of EDTA, urine samples were gathered and heavy metal concentrations were measured. We used validated and accredited methods according to International Organization for Standardization and International Electrotechnical Commission standard 17025; the indicated criteria were applied. Inductively coupled plasma mass spectrometry was implemented to measure the mentioned heavy metals’ concentrations; this measurement process was executed at Taipei Veterans General Hospital Department of Clinical Toxicology.

### 2.4 Statistical analysis

In our executed study, we implemented SPSS Statistics (version 20; IBM Corp., Armonk, NY) to perform our statistical analyses. We appraised the strength of the association between heavy metal concentrations and the indicated variables by employing Spearman correlation coefficient. To perform *P*-value adjustment, we employed the false discovery rate, which was estimated using the Benjamini–Hochberg procedure. In addition, we assessed the differences between AIS patient variables with respect to heavy metal concentrations by executing an independent-samples Mann–Whitney *U* test. We deemed a *P*-value of <.05 as signifying statistical significance. Moreover, the derived analysis data are presented herein as mean ± SD.

## 3 Results

### 3.1 Participants’ baseline characteristics

Thirty-three patients with AIS were enrolled for participation in this study for the period between August 2011 and March 2013. In the study population, 81.8% and 18.2% of the enrollees were men and women, respectively (average age: 58 years). Table 1 presents the baseline data of the participants. We assessed patient variables such as age, sex, BMI, hypertension, diabetes, hyperlipidemia, smoking, alcohol consumption, stroke type, and stroke severity. Furthermore, we measured serum and urine concentrations of Hg, Cd, As, and Pb and performed comparisons to determine the correlations between such metal concentrations and the aforementioned variables.

**Table 1:** Participants’ (ie, patients with acute ischemic stroke) baseline demographic data.

Variables	No. (%)
Gender	
Female	6 (18.2%)
Male	27 (81.8%)
Age (y/o, *mean* *±* *SD*)	57.7 ± 10.5
BMI (*mean* *±* *SD*)	25.7 ± 3.8
Hypertension	
Yes	27 (81.8%)
No	6 (18.2%)
Diabetes	
Yes	13 (39.4%)
No	20 (60.6%)
Hyperlipidemia	
Yes	30 (90.9%)
No	3 (9.1%)
Smokes	
Yes	17 (51.5%)
No	16 (48.5%)
Alcohol consumption	
Yes	9 (27.3%)
No	24 (72.7%)
Stroke type	
LAA	15 (45.5%)
Non-LAA	18 (54.5%)
Stroke severity	
<6	19 (57.6%)
≥6	14 (42.4%)

BMI = body mass index, LAA = large artery atherosclerosis.

### 3.2 Associations between heavy metal concentrations and blood chemistry, BMI, hematology, and blood pressure in participants

Using Spearman correlation coefficient, we gauged the associations between serum concentrations of heavy metal and BMI, blood pressure, blood chemistry, and hematology in the participants. As shown in Table 2, we revealed that serum Pb (S-Pb) concentrations had a positive correlation with serum creatinine (*P* < .041), red blood cells (*P* < .022), hemoglobin (*P* < .003), and hematocrit (*P* < .004). However, S-Pb concentrations had a negative correlation with serum sodium (*P* < .02). Serum Cd (S-Cd) concentrations had a positive correlation with serum creatinine (*P* < .009) and hemoglobin (*P* < .046) but a negative correlation with serum glycosylated hemoglobin (HbA1c, *P* < .042) and fasting blood sugar (*P* < .03) levels. Nevertheless, our results indicated no associations of serum Hg (S-Hg) and serum AS (S-As) with blood chemistry or hematology. Furthermore, we identified urine heavy metal concentrations to be associated with BMI, blood pressure, blood chemistry, and hematology in the participants (Table 3). Spearman's correlation demonstrated that urine Pb (U-Pb) concentrations were positively correlated with serum hemoglobin (*P* < .039). Additionally, a positive correlation was noted between urine Hg (U-Hg) concentrations and C-reactive protein in serum (*P* < .017). However, U-Hg concentrations had a negative correlation with platelets in serum (*P* < .029). Concentrations of Cd (U-Cd) in urine had a negative correlation with HbA1c in serum (*P* < .004). Urine As (U-As) concentrations were not associated with blood chemistry or hematology in the participants.

**Table 2:** Association of serum heavy metal concentrations with the blood pressure, BMI, hematology, and blood chemistry of patients with acute ischemic stroke.

	S-Pb (μg/L)			S-Hg (μg/L)			S-As (μg/L)			S-Cd (μg/L)		
Variables	No.	*r* ^∗^	*P*-value	No.	*r* ^∗^	*P*-value	No.	*r* ^∗^	*P*-value	No.	*r* ^∗^	*P*-value
BMI	33	0.153	.396	30	0.258	.168	33	0.293	.098	33	0.138	.445
Blood pressure												
SBP	30	−0.038	.843	27	−0.016	.937	30	0.247	.189	30	−0.099	.601
DBP	30	0.038	.840	27	0.180	.369	30	0.099	.604	30	−0.016	.932
Blood chemistry												
HbA1c	26	−0.064	.755	23	−0.175	.424	26	0.021	.918	26	−0.401	**.042**
FBG	31	−0.024	.897	28	−0.119	.545	31	0.263	.152	31	−0.390	**.030**
TG	32	0.136	.457	29	0.174	.365	32	−0.099	.588	32	0.070	.703
T-CHO	33	−0.029	.875	30	0.180	.342	33	0.090	.619	33	0.033	.855
HDL-C	33	−0.138	.445	30	0.234	.213	33	0.122	.498	33	0.192	.284
LDL-C	33	0.042	.814	30	−0.059	.759	33	−0.009	.960	33	−0.120	.505
BUN	33	−0.090	.617	30	0.210	.265	33	0.067	.713	33	0.030	.869
Crea	33	0.358	**.041**	30	0.233	.215	33	−0.046	.798	33	0.445	**.009**
Na	33	−0.403	**.020**	30	−0.118	.536	33	0.189	.292	33	−0.202	.260
K	33	−0.206	.251	30	0.276	.140	33	−0.044	.810	33	−0.006	.973
GOT	30	−0.166	.381	27	0.246	.216	30	0.173	.360	30	0.200	.289
GPT	33	−0.101	.575	30	0.166	.381	33	−0.083	.647	33	−0.066	.715
CRP	30	0.280	.134	27	0.001	.995	30	−0.081	.672	30	0.138	.467
UA	30	0.233	.215	27	0.347	.076	30	0.089	.641	30	0.283	.129
ESR	33	−0.097	.592	30	−0.154	.418	33	0.121	.501	33	−0.256	.150
Hematology												
WBC	33	0.231	.197	30	0.052	.786	33	0.014	.937	33	0.181	.314
RBC	33	0.399	**.022**	30	−0.169	.373	33	−0.271	.127	33	0.133	.459
HGB	33	0.507	**.003**	30	0.079	.680	33	−0.114	.527	33	0.350	**.046**
HCT	33	0.484	**.004**	30	0.084	.659	33	−0.141	.434	33	0.335	.057
PLT	33	0.135	.454	30	0.008	.967	33	−0.229	.200	33	−0.086	.636

BMI = body mass index, BUN = blood urea nitrogen, Crea = serum creatinine, CRP = C-reactive protein, DBP = diastolic blood pressure, ESR = erythrocyte sedimentation rate, FBG = fasting blood sugar, TG = triglyceride, GOT = glutamic oxaloacetic transaminase, GPT = glutamic pyruvic transaminase, HbA1c = glycosylated hemoglobin, HCT = hematocrit, HDL-C = high density lipoprotein cholesterol, HGB = hemoglobin, LDL-C = low density lipoprotein cholesterol, PLT = platelets, RBC = red blood cells, S-As = serum arsenic, SBP = systolic blood pressure, S-Cd = serum cadmium, S-Hg = serum mercury, S-Pb = serum lead, T-CHO = cholesterol, UA = uric acid, WBC = white blood cells.

∗ *r*, correlation coefficients and *P*-values were estimated using Spearman correlation coefficient. The significance of bold values is a *P*-value of <.05.

**Table 3:** Association of urine heavy metal concentrations with blood pressure, BMI, hematology, and blood chemistry in participants.

	U-Pb (μg/L)			U-Hg (μg/L)			U-As (μg/L)			U-Cd (μg/L)		
Variables	No.	*R* ^∗^	*P*-value	No.	*R* ^∗^	*P*-value	No.	*R* ^∗^	*P*-value	No.	*R* ^∗^	*P*-value
BMI	33	0.134	.457	33	0.062	.732	33	0.318	.071	33	−0.102	.572
Blood pressure												
SBP	30	−0.005	.979	30	−0.014	.943	30	0.052	.786	30	0.016	.933
DBP	30	−0.047	.805	30	−0.090	.636	30	−0.016	.932	30	−0.065	.734
Blood chemistry												
HbA1c	26	−0.322	.109	26	−0.285	.158	26	0.015	.943	26	−0.543	**.004**
FBG	31	−0.081	.665	31	−0.065	.728	31	0.286	.119	31	−0.289	.115
TG	32	−0.170	.352	32	0.186	.307	32	−0.155	.396	32	0.026	.888
T-CHO	33	0.066	.714	33	−0.008	.963	33	0.118	.512	33	0.148	.411
HDL-C	33	0.091	.615	33	0.167	.354	33	−0.130	.471	33	0.330	.060
LDL-C	33	0.167	.354	33	−0.238	.182	33	0.187	.298	33	−0.128	.479
BUN	33	−0.113	.530	33	0.108	.550	33	0.112	.534	33	0.220	.219
Crea	33	0.157	.383	33	0.339	.054	33	−0.012	.947	33	0.076	.685
Na	33	−0.305	.084	33	−0.321	.068	33	0.056	.757	33	−0.125	.490
K	33	−0.133	.461	33	−0.112	.535	33	−0.072	.692	33	−0.048	.793
GOT	30	0.185	.329	30	0.344	.062	30	0.094	.621	30	0.328	.077
GPT	33	−0.170	.345	33	−0.059	.745	33	−0.040	.824	33	−0.135	.453
CRP	30	0.278	.137	30	0.433	**.017**	30	−0.061	.747	30	0.356	.053
UA	30	0.287	.124	30	0.091	.631	30	0.004	.981	30	0.049	.797
ESR	33	0.131	.469	33	0.195	.277	33	0.029	.872	33	0.277	.119
Hematology												
WBC	33	0.020	.912	33	−0.093	.608	33	−0.187	.298	33	−0.213	.234
RBC	33	0.024	.895	33	−0.137	.447	33	−0.184	.305	33	−0.287	.105
HGB	33	0.360	**.039**	33	0.022	.901	33	0.031	.863	33	−0.031	.865
HCT	33	0.330	.061	33	0.016	.930	33	0.031	.864	33	−0.035	.845
PLT	33	−0.023	.899	33	−0.380	**.029**	33	−0.194	.281	33	−0.325	.065

BMI = body mass index, BUN = blood urea nitrogen, Crea = serum creatinine, CRP = C-reactive protein, DBP = diastolic blood pressure, ESR = erythrocyte sedimentation rate, FBG = fasting blood sugar, TG = triglyceride, GOT = glutamic oxaloacetic transaminase, GPT = glutamic pyruvic transaminase, HbA1c = glycosylated hemoglobin, HCT = hematocrit, HDL-C = high-density lipoprotein cholesterol, HGB = hemoglobin, LDL-C = low-density lipoprotein cholesterol, PLT = platelets, RBC = red blood cells, S-As = serum arsenic, SBP = systolic blood pressure, S-Cd = serum cadmium, S-Hg = serum mercury, S-Pb = serum lead, T-CHO = cholesterol, UA = uric acid, WBC = white blood cells.

∗ *r*, correlation coefficients and *P-*values were estimated using Spearman correlation coefficient. The significance of bold values is a *P*-value of <.05.

### 3.3 Smoking, alcohol consumption, and hypertension increased heavy metal elevation in participants

We gauged differences in heavy metal concentrations between the different patient variables. Participants who smoked had significantly higher concentrations of S-Pb (25.3 ± 8.5 μg/L vs 17.5 ± 6.3 μg/L, *P* < .011) and S-Cd (1.0 ± 0.5 μg/L vs 0.6 ± 0.2 μg/L, *P* < .002) compared with participants who did not smoke (Table 4). Furthermore, participants who consumed alcohol had significantly higher concentrations of S-Pb (29.5 ± 8.6 μg/L vs 18.5 ± 6.1 μg/L, *P* < .002), S-Cd (1.2 ± 0.6 μg/L vs. 0.6 ± 0.3 μg/L, *P* < .004), U-Pb (14.3 ± 5.7 μg/L vs 10.1 ± 7.8 μg/L, *P* < .015), and U-Hg (1.0 ± 0.5 μg/L vs 0.7 ± 0.7 μg/L, *P* < .039) compared with participants who did not consume alcohol (Table 4). Participants with hypertension had significantly higher concentrations of S-As (6.1 ± 5.9 μg/L vs 3.0 ± 0.3 μg/L, *P* < .04) than did those without hypertension.

**Table 4:** Comparison of serum and urine heavy metal concentrations in participants (ie, patients with acute ischemic stroke) who smoked, consumed alcohol, and had hypertension.

	Smokes			Alcohol consumption			Hypertension		
	Yes (n = 17)	No (n = 16)		Yes (n = 9)	No (n = 24)		Yes (n = 27)	No (n = 6)	
Variables	Mean ± SD	Mean ± SD	*P*-value	Mean ± SD	Mean ± SD	*P*-value	Mean ± SD	Mean ± SD	*P*-value
S-Pb (μg/L)	25.3 ± 8.5	17.5 ± 6.3	**.011**	29.5 ± 8.6	18.5 ± 6.1	**.002**	21.9 ± 8.5	19.6 ± 8.0	.455
S-Hg (μg/L)	(n = 15)	(n = 15)	.756	(n = 8)	(n = 22)	.174	(n = 25)	(n = 5)	.522
	6.5 ± 4.0	6.2 ± 4.6		8.0 ± 4.9	5.7 ± 3.9		6.6 ± 4.5	5.0 ± 2.5	
S-As (μg/L)	4.7 ± 3.3	6.5 ± 7.1	.313	6.3 ± 3.7	5.3 ± 6.0	.225	61. ± 5.9	3.0 ± 0.3	**.040**
S-Cd (μg/L)	1.0 ± 0.5	0.6 ± 0.2	**.002**	1.2 ± 0.6	0.6 ± 0.3	**.004**	0.8 ± 0.5	0.9 ± 0.4	.304
U-Pb (μg/L)	12.6 ± 9.0	9.9 ± 5.3	.407	14.3 ± 5.7	10.1 ± 7.8	**.015**	12.0 ± 8.0	7.9 ± 2.3	.243
U-Hg (μg/L)	0.8 ± 0.5	0.7 ± 0.8	.517	1.0 ± 0.5	0.7 ± 0.7	**.039**	0.8 ± 0.7	0.4 ± 0.3	.208
U-As (μg/L)	60.2 ± 83.6	45.8 ± 30.8	.801	77.4 ± 109.8	44.2 ± 32.5	.544	59.5 ± 68.3	24.9 ± 11.7	.084
U-Cd (μg/L)	2.4 ± 2.8	1.6 ± 1.1	.914	3.4 ± 3.4	1.5 ± 1.2	.115	2.3 ± 2.3	0.9 ± 0.4	.056

S-As = serum arsenic, S-Cd = serum cadmium, S-Hg = serum mercury, S-Pb = serum lead, U-As = urine arsenic, U-Cd = urine cadmium, U-Hg = urine mercury, U-Pb = urine lead. The significance of bold values is a *P*-value of <.05.

Concentrations of U-Pb, S-Pb, U-Hg, S-Hg, S-As, S-Cd, U-As, and U-Cd were not significantly different between the sexes (Table S1, Supplemental Digital Content); sex is a nonmodifiable risk factor for AIS. However, between modifiable AIS risk factors, including diabetes (Table S2, Supplemental Digital Content), hyperlipidemia (Table S3, Supplemental Digital Content), stroke type (Table S4, Supplemental Digital Content), and stroke severity (Table S5, Supplemental Digital Content), we noted no significant differences in S-Hg, S-Pb, S-As, U-Hg, S-Cd, U-Pb, U-As, or U-Cd concentrations. Next, we conducted a Spearman correlation analysis of the concentrations of each indicated metal in the serum and urine of the study participants, all of whom resided in Southern Taiwan (Table 5). We found serum Pb to be significantly and positively correlated with serum Cd, urine Pb, and urine Cd. We additionally noted serum Hg to be significantly and positively correlated with serum As, serum Cd, urine Hg, and urine Cd. The results also indicated serum As to be significantly and positively correlated with Hg and As in urine. In addition, we determined serum Cd to be significantly and positively correlated with Pb and Cd in urine. Moreover, urine Pb was noted to be significantly and positively correlated with Hg, As, and Cd in urine. Finally, we observed urine Hg to be significantly and positively correlated with urine As and urine Cd.

**Table 5:** Correlation coefficients (*r*) of the concentration of each heavy metal in serum and urine.

Variables	S-Pb (μg/L)	(n = 30) S-Hg (μg/L)	S-As (μg/L)	S-Cd (μg/L)	U-Pb (μg/L)	U-Hg (μg/L)	U-As (μg/L)	U-Cd (μg/L)
S-Pb (μg/L)	–							
(n = 30)S-Hg (μg/L)	*r* = 0.204 *P* = .278	–						
S-As (μg/L)	r = 0.081 *P* = .655	**r** **=** **0.610** ***P*** **<** **.001**	–					
S-Cd (μg/L)	***r*** **=** **0.673** ***P*** **<** **.001**	***r*** **=** **0.510** ***P*** **=** **.004**	*r* = 0.153 *P* = .395	–				
U-Pb (μg/L)	***r*** **=** **0.679** ***P*** **<** **.001**	*r* = 0.313 *P* = .093	*r* = 0.342 *P* = .051	***r*** **=** **0.486** ***P*** **=** **.006**	–			
U-Hg (μg/L)	*r* = 0.233 *P* = .193	***r*** **=** **0.384** ***P*** **=** **.036**	***r*** **=** **0.345** ***P*** **=** **.050**	*r* = 0.140 *P* = .437	***r*** **=** **0.456** ***P*** **=** **.008**	–		
U-As (μg/L)	*r* = 0.147 *P* = .413	*r* = 0.295 *P* = .114	***r*** **=** **0.762** ***P*** **<** **.001**	*r* = 0.009 *P* = .962	***r*** **=** **0.483** ***P*** **=** **.004**	***r*** **=** **0.345** ***P*** **=** **.050**	–	
U-Cd (μg/L)	***r*** **=** **0.394** ***P*** **=** **.023**	***r*** **=** **0.406** ***P*** **=** **.026**	*r* = 0.256 *P* = .150	***r*** **=** **0.479** ***P*** **=** **.005**	***r*** **=** **0.635** ***P*** **<** **.001**	***r*** **=** **0.521** ***P*** **=** **.002**	*r* = 0.243 *P* = .173	–

Correlation coefficients and *P*-values were estimated using Spearman rank correlation coefficient. The significance of bold values is a *P*-value of <.05.

## 4 Discussion

The economy of southern Taiwan mainly depends on heavy industry. Occupational exposure to several harmful materials, including biological substances, heavy metals, benzene, and chemicals, adversely affects human health through the development of certain conditions, such as cancer and cardiovascular, hematologic, reproductive, immunologic, and neurodegenerative diseases. Many researchers have demonstrated exposure to certain heavy metals to be associated with significant increases in cerebrovascular event (eg, stroke) prevalence. Therefore, occupational exposure to heavy metals could damage the health of those residing in southern Taiwan. In this study, we investigated the associations between the concentrations of lead, As, Cd, and Hg in our participants (ie, AIS patients) and AIS risk factors, which were classified as either nonmodifiable (age and sex) or modifiable (hypertension, cigarette smoking, diabetes, hyperlipidemia, and alcohol consumption). We noted S-Pb and S-Cd concentrations to exhibit a positive correlation with alcohol consumption and smoking. In addition, we noted U-Pb and U-Hg concentrations to exhibit a positive correlation with alcohol consumption. Finally, we determined S-As concentrations to exhibit a positive correlation with hypertension.

Recently, research has demonstrated that some sampled wines contained heavy metals, raising contamination concerns. Pb from environmental (soil composition, climate, and region) and anthropogenic sources (winery equipment, industrial emissions, chemical sprays, fertilizers, and metal-based pesticides)^[21]^ was detected in 58% of representative wine samples (ie, 65 samples) obtained from the four top wine production states in the United States.^[22]^ Furthermore, the amount of Pb in a cigarette is approximately 1.2 μg, 6% of which is present in mainstream smoke.^[23]^ Smoking might lead to impaired endothelium-dependent relaxation induced by angiotensin II and cause the overproduction of superoxide, both of which can lead to the development of cardiovascular diseases.^[24]^ Pb accumulation can result in peripheral arterial diseases.^[14]^ Cd and Pb exposure result in a significant increase in stroke risk.^[7]^ In our study, patients with AIS who smoked or consumed alcohol were determined to exhibit significantly higher concentrations of S-Pb and S-Cd compared with those who did not. Moreover, cumulative exposure can increase the Pb tissue burden; this has adverse health effects at specific exposure levels, including renal, hematologic, nervous, and reproductive system effects.^[25]^ We also observed that S-Pb concentrations were positively correlated with hematology (red blood cells, hemoglobin, hematocrit) and serum creatinine, whereas U-Pb concentrations were positively correlated with hemoglobin in patients with AIS. However, high exposure levels of Pd or Cd resulted in reductions in hematocrit, hemoglobin, and red blood cell count measurements in animal models.^[26]^

Of all heavy metals, exposure to Hg is the most harmful,^[27]^ such exposure can alter the retention and distribution of other heavy metals. Hg physiological role in the metabolism of humans is unknown, and humans lack the ability to actively excrete it.^[28]^ Regarding Hg toxicity, clinical manifestations include atherosclerosis, coronary heart disease, hypertension, cerebrovascular events, myocardial infarction, proteinuria, and renal dysfunction.^[15]^ Even at low concentrations, chronic exposure to Hg might reduce nitric oxide bioavailability, in addition to increasing oxidative stress and inflammation. Therefore, long-term Hg exposure can induce endothelial dysfunction and consequently increase cardiovascular as well as cerebrovascular disease risk.^[29]^ Furthermore, we observed that U-Hg concentrations were positively correlated with serum C-reactive protein but negatively correlated with platelet counts. Cardiovascular outcomes of Hg exposure, including stroke, are alleviated by the concomitant consumption of fish containing Omega-3 oils.^[27]^ However, the findings derived from our southern Taiwan–based study as well as a study involving a US cohort^[30]^ do not support the hypothesis of AIS incidence and Hg being associated in a population with an exposure level that is moderate or low. A previously executed systematic review and meta-analysis that involved the use of nonoverlapping data of roughly 350 000 study participants (number of studies: 37) suggested that Hg and As were not significantly associated with stroke risk.^[7]^

Cd is highly toxic, with its half-life being nearly one to three decades; its accumulation in the body of humans is most notable in the kidneys and liver.^[31]^ Epidemiologic studies have used 24-h concentrations of U-Cd as a biological marker of lifetime exposure, and S-Cd concentrations have been used as an indicator of exposure in recent months.^[32]^ Cd exposure mainly occurs through the consumption of contaminated water and food as well as through the inhalation of smoke and polluted air.^[33]^ A meta-analysis of epidemiological investigations indicated that Cd is a risk factor for atherosclerosis.^[34]^ In our cohort, S-Cd concentrations were positively correlated with impaired serum creatinine; this finding is consistent with those of Japanese studies, which have reported that high Cd exposure levels increased the incidence of cerebral infarction and renal diseases.^[35,36]^ A study that applied data derived from the 1999 to 2006 National Health and Nutrition Examination Survey demonstrated that after adjustment for cardiovascular risk factors as well as for demographic data, a 50% increase in Cd concentrations was accompanied by a 35% increase in the prevalence of stroke.^[37]^ In an analysis of heavy metal associations, higher concentrations of As and Cd in plasma were positively associated with a higher prevalence of AIS in China.^[10]^ Furthermore, smoking contributed to increased S-Cd concentration in non-Hispanic Asian adults,^[38]^ which is consistent with the increased levels observed in smokers in our cohort. Additionally, in the final phase of treatment for Cd exposure, ethanol administration was reported to aggravate Cd accumulation in the liver and kidney and to reduce the urinary and fecal excretion of Cd, regardless of the Cd treatment level.^[39]^ This indicates that alcohol consumption may affect Cd accumulation; however, further research on this is required. Certain study limitations must be noted. First, our single-institute study involved a small sample size. Second, some covariates (eg, smoking and alcohol consumption) were self-reported, introducing the possibility of recall bias. Third, heavy metals exposure time has remained unclear.

## 5 Conclusions

Our results indicate the potential associations between multiple heavy metals and AIS risk in a population in southern Taiwan. Specifically, we demonstrated a potential association between hypertension and high concentrations of S-As in patients with AIS. Smoking may have caused patients with AIS to have high concentrations of S-Cd and S-Pb. Alcohol consumption may have caused patients with AIS to have high concentrations of S-Pb, S-Cd, U-Pb, and U-Hg. This prospective study provides evidence that smoking and alcohol consumption increased the levels up of multiple heavy metals in a southern Taiwan population of patients with AIS. Prospective studies involving larger sample sizes are needed for the confirmation of our findings.

## Acknowledgments

We thank Huei-Han Liou and Hui-Yu Chang for their technical assistance and invaluable input on this manuscript.

## Author contributions

**Conceptualization:** Hsin-Hung Chen.

**Data curation:** Yi-Ting Hsu

**Methodology:** Cheng-Chang Yen

**Project administration:** Ching-Huang Lin

**Resources:** Ching-Huang Lin

**Supervision:** Ching-Jiunn Tseng

**Validation:** Cheng-Chang Yen

**Writing – original draft:** Cheng-Chang Yen, Hsin-Hung Chen

**Writing – review & editing:** Ching-Huang Lin

## Supplementary Material

Supplemental Digital Content

## Supplementary Material

Supplemental Digital Content

## Supplementary Material

Supplemental Digital Content

## Supplementary Material

Supplemental Digital Content

## Supplementary Material

Supplemental Digital Content
