# Supplementary material for: Effects of heavy metals in acute ischemic stroke patients: A cross-sectional study
Source: Medicine (Baltimore). 2022 Mar 4;101(9):e28973. doi: 10.1097/MD.0000000000028973 (PMC8896421; doi:10.1097/MD.0000000000028973)
Supplement: Supplemental Digital Content [file medi-101-e28973-s001.doc]

**Effects of** **Heavy Metals in Acute Ischemic Stroke Patients: A cross-sectional study**

Cheng-Chang Yen, M.D.^a,b^, Hsin-Hung Chen, Ph.D.^c^, Yi-Ting Hsu, Ms.^a^, Ching-Jiunn Tseng, M.D., Ph.D.^c,d^, and Ching-Huang Lin, M.D.,Ph.D.^a,e,f^

^a^Section of Neurology, Kaohsiung Veterans General Hospital, No. 386, Ta-Chung 1st Rd., Kaohsiung, 813414, Taiwan

^b^Department of Electrical Engineering, Southern Taiwan University of Science and Technology, No. 1, Nan-Tai Street, Yongkang Dist., Tainan, 71005, Taiwan

^c^Department of Medical Education and Research, Kaohsiung Veterans General Hospital, No. 386, Ta-Chung 1st Rd., Kaohsiung, 813414, Taiwan

^d^Department of Medical Research, China Medical University Hospital, China Medical University, No. 2, Yude Rd., Taichung, 40447, Taiwan

^e^Department of Biological Sciences, National Sun Yat-Sen University, 70 Lienhai Rd., Kaohsiung 80424, Taiwan

^f^Department of Physical Therapy, Shu-Zen Junior College of Medicine and Management, No.452, Huanqiu Rd. Luzhu Dist., Kaohsiung, 82144, Taiwan

CCY and HHC have contributed equally to this article.

**Corresponding author:**

Ching-Huang Lin, MD, PhD

Section of Neurology, Kaohsiung Veterans General Hospital, Kaohsiung Veterans General Hospital, No. 386, Ta-Chung 1st Rd., Kaohsiung, 813414, Taiwan

Tel: +886-7-3422121 ext.2034; Fax: +886-7-3468056

E-mail: [chlin2524@vghks.gov.tw](mailto:chlin2524@vghks.gov.tw)

| **Supplemental table 1. Comparison of serum and urine heavy metal levels for participants between female and male** | | | | |
| --- | --- | --- | --- | --- |
| **Variables** | **Male**  **(n = 27)** |  | **Female**  **(n = 6)** | ***P*** |
|  | Mean ± SD |  | Mean ± SD |  |
| S-Pb (μg/L) | 22.2 ± 8.6 |  | 18.3 ± 7.0 | 0.327 |
| S-Hg (μg/L) | (n=24) |  | (n=6) | 0.836 |
|  | 6.1 ± 3.6 |  | 7.3 ± 6.7 |  |
| S-As (μg/L) | 5.6 ± 5.9 |  | 5.6 ± 3.0 | 0.304 |
| S-Cd (μg/L) | 0.8 ± 0.5 |  | 0.6 ± 0.2 | 0.455 |
| U-Pb (μg/L) | 11.5 ± 7.5 |  | 10.2 ± 7.5 | 0.455 |
| U-Hg (μg/L) | 0.7 ± 0.5 |  | 0.9 ± 1.3 | 0.427 |
| U-As (μg/L) | 54.6 ± 69.0 |  | 46.9 ± 27.5 | 0.674 |
| U-Cd (μg/L) | 2.0± 2.3 |  | 2.0 ± 1.3 | 0.455 |
| *Note.* S-Pb= serum lead; S-Hg= serum mercury; S-As= serum arsenic; S-Cd=  serum cadmium; U-Pb= urine lead; U-Hg= urine mercury; U-As= urine arsenic; U-Cd= urine cadmium | | | | |
