# Supplementary material for: Effects of heavy metals in acute ischemic stroke patients: A cross-sectional study
Source: Medicine (Baltimore). 2022 Mar 4;101(9):e28973. doi: 10.1097/MD.0000000000028973 (PMC8896421; doi:10.1097/MD.0000000000028973)
Supplement: Supplemental Digital Content [file medi-101-e28973-s004.doc]

**Effects of** **Heavy Metals in Acute Ischemic Stroke Patients: A cross-sectional study**

Cheng-Chang Yen, M.D.^a,b^, Hsin-Hung Chen, Ph.D.^c^, Yi-Ting Hsu, Ms.^a^, Ching-Jiunn Tseng, M.D., Ph.D.^c,d^, and Ching-Huang Lin, M.D.,Ph.D.^a,e,f^

^a^Section of Neurology, Kaohsiung Veterans General Hospital, No. 386, Ta-Chung 1st Rd., Kaohsiung, 813414, Taiwan

^b^Department of Electrical Engineering, Southern Taiwan University of Science and Technology, No. 1, Nan-Tai Street, Yongkang Dist., Tainan, 71005, Taiwan

^c^Department of Medical Education and Research, Kaohsiung Veterans General Hospital, No. 386, Ta-Chung 1st Rd., Kaohsiung, 813414, Taiwan

^d^Department of Medical Research, China Medical University Hospital, China Medical University, No. 2, Yude Rd., Taichung, 40447, Taiwan

^e^Department of Biological Sciences, National Sun Yat-Sen University, 70 Lienhai Rd., Kaohsiung 80424, Taiwan

^f^Department of Physical Therapy, Shu-Zen Junior College of Medicine and Management, No.452, Huanqiu Rd. Luzhu Dist., Kaohsiung, 82144, Taiwan

CCY and HHC have contributed equally to this article.

**Corresponding author:**

Ching-Huang Lin, MD, PhD

Section of Neurology, Kaohsiung Veterans General Hospital, Kaohsiung Veterans General Hospital, No. 386, Ta-Chung 1st Rd., Kaohsiung, 813414, Taiwan

Tel: +886-7-3422121 ext.2034; Fax: +886-7-3468056

E-mail: [chlin2524@vghks.gov.tw](mailto:chlin2524@vghks.gov.tw)

| **Supplemental table 4. Comparison of serum and urine heavy metal levels for participants between stroke type** | | | | |
| --- | --- | --- | --- | --- |
| **Variables** | **LAA**  **(n = 15)** |  | **Non-LAA**  **(n = 18)** | ***P*** |
|  | Mean ± SD |  | Mean ± SD |  |
| S-Pb (μg/L) | 23.1 ± 8.6 |  | 20.2 ± 8.2 | 0.294 |
| S-Hg (μg/L) | (n=14) |  | (n=16) | 0.212 |
|  | 6.0 ± 4.7 |  | 6.7 ± 4.0 |  |
| S-As (μg/L) | 4.0 ± 2.4 |  | 6.9 ± 6.9 | 0.138 |
| S-Cd (μg/L) | 0.9 ± 0.6 |  | 0.7 ± 0.3 | 0.492 |
| U-Pb (μg/L) | 12.0 ± 6.9 |  | 10.7 ± 8.0 | 0.470 |
| U-Hg (μg/L) | 0.9 ± 0.9 |  | 0.6 ± 0.4 | 0.942 |
| U-As (μg/L) | 38.6 ± 27.7 |  | 65.4 ± 80.9 | 0.311 |
| U-Cd (μg/L) | 2.8 ± 2.9 |  | 1.5 ± 1.0 | 0.470 |
| *Note.* S-Pb= serum lead; S-Hg= serum mercury; S-As= serum arsenic; S-Cd=  serum cadmium; U-Pb= urine lead; U-Hg= urine mercury; U-As= urine arsenic; U-Cd= urine cadmium. | | | | |
